# Typical presentation of autosomal recessive oculocutaneous albinism in two siblings

**DOI:** 10.3205/oc000249

**Published:** 2025-04-10

**Authors:** Prateek Nishant, Naila Aftab, Bhawesh Saha, Amit Raj

**Affiliations:** 1 Department of Ophthalmology, All India Institute of Medical Sciences, Patna, India

**Keywords:** fovea centralis, hypomelanosis, iris, low vision, melanin

## Abstract

**Objective:**

We report the case history and clinical findings in two siblings, a 13-year-old male and a 10-year-old female, who presented with complaints of poor vision since childhood. Both children had blonde hair and depigmented skin.

**Methods:**

Ocular examination revealed white eyebrows, white eyelashes, diminished vision in all eyes, hypochromic irides and pendular nystagmus. On dilated fundus examination, hypopigmented fundi with conspicuously visible choroidal vessels were noted. No foveolar reflex could be discerned and spectral domain optical coherence tomography (SD-OCT) of the macula showed an absence of the foveal pit in all four eyes. On pedigree charting the subjects were the 2^nd^ and 3^rd^ offspring of a non-consanguineous married couple. One of the mother’s siblings and one of the grandmother’s siblings also had a similar disorder.

**Results:**

The poor definition of the foveal pit at the centre of the macula, i.e. foveal hypoplasia, accounted for poor visual acuity and nystagmus. Both cases had no syndromic associations. Spectacle correction was prescribed to both children, and low-vision aids and sun protection advised.

**Conclusion:**

Oculocutaneous albinism (OCA) represents a range of inherited, congenital disorders of hypomelanosis, involving the skin, hair, and eyes with an estimated prevalence of 1 in 17,000 cases. Affected children suffer severe visual disability while early identification may potentially mitigate it, hence there is need to sensitize primary care practitioners regarding the general symptoms of OCA.

## Introduction

Absence of melanin pigmentation results in albinism which is classically characterised in two groups according to the distribution of the hypomelanosis – oculocutaneous albinism (OCA) and ocular albinism. Non-syndromic OCA represents a range of inherited, congenital disorders of hypomelanosis, involving the skin, hair and eyes, with an estimated prevalence of 1 in 17,000 to 1 in 20,000 [1], [2], [3]. There are at least four main types and four minor types of non-syndromic albinism, differing in the genes mutated and in few clinical features [4]. Here, we exhibit the typical presentation of OCA in two siblings.

## Case description

**Figure 1:**
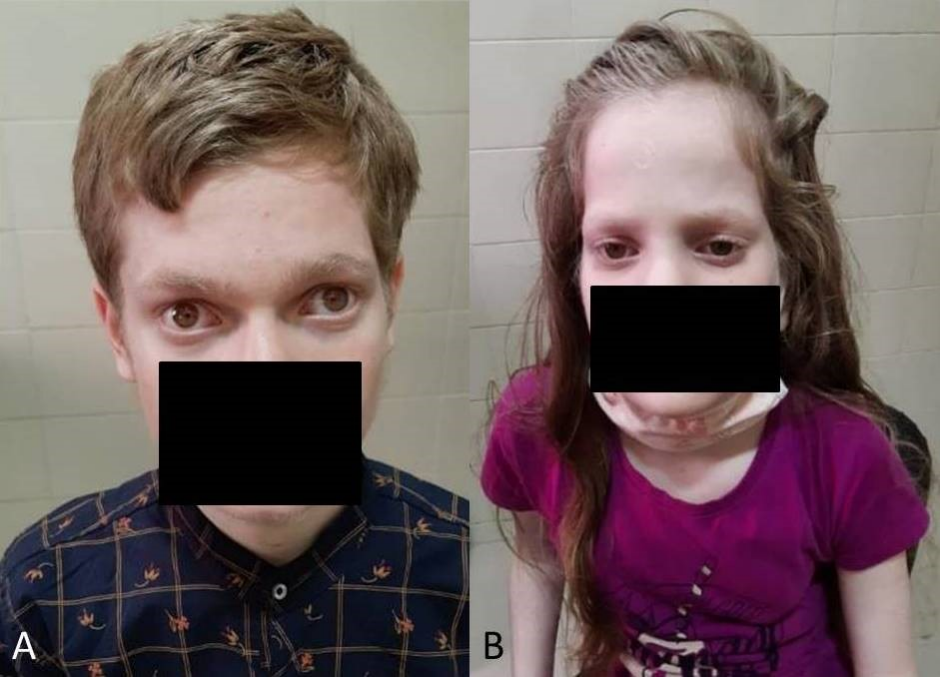
Full face images of sibling 1 (A) and sibling 2 (B), respectively

Two Asian Indian siblings – a 13-year-old male (sibling 1, Figure 1A (Fig. 1)) and a 10-year-old female (sibling 2, Figure 1B (Fig. 1)) – presented with complaints of poor vision in both eyes since early childhood. There was no history of use of spectacles. Presenting uncorrected visual acuity was poorer than 6/60 in both eyes (Table 1 (Tab. 1)). On examination, both children had blonde hair, eyebrows, and eyelashes, depigmented skin, as well as pendular nystagmus. The boy had alternating exotropia of 15 degrees.

**Figure 2:**
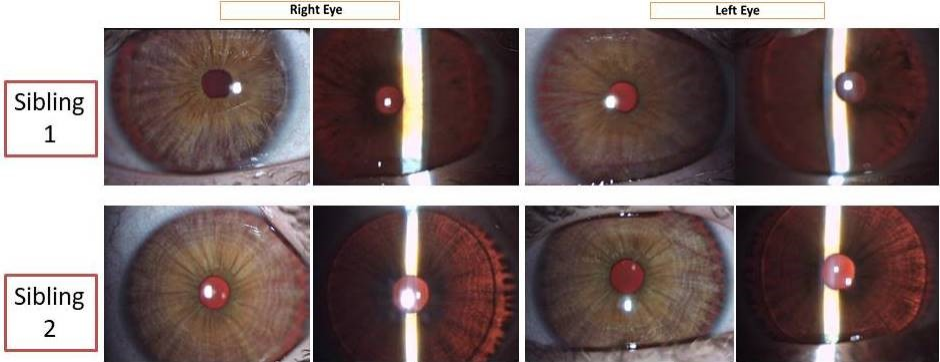
Anterior segment photographs showing hypochromic irides in diffuse illumination, and iris transillumination

**Figure 3:**
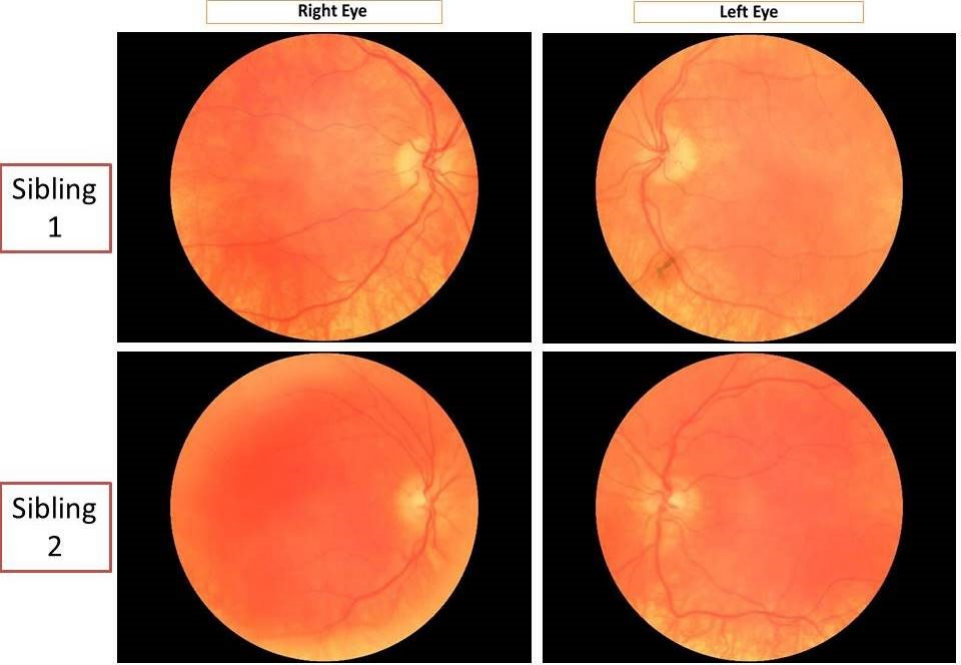
Fundus photographs showing hypopigmented fundi with conspicuous choroidal vessels

Anterior segment examination of both children showed hypochromic irides with transillumination defects in both eyes (Figure 2 (Fig. 2)). The fundi were hypopigmented with conspicuous choroidal vessels and no foveolar reflex (Figure 3 (Fig. 3)).

Stereoacuity testing and cycloplegic retinoscopy were done followed by post-mydriatic test. The results are summarised in Table 1 (Tab. 1).

**Figure 4:**
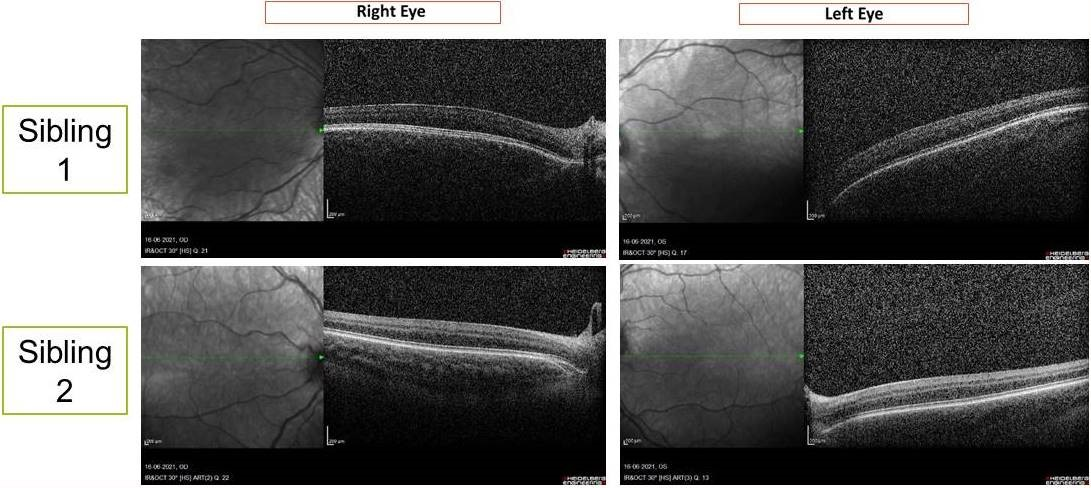
Spectral domain optical coherence tomography (horizontal line scans) through the approximate position of the fovea showing no foveal dip in any of the eyes

Spectral domain optical coherence tomography of the macula of both children showed absence of foveal pit suggestive of foveal hypoplasia in both eyes (Figure 4 (Fig. 4)). 

**Figure 5:**
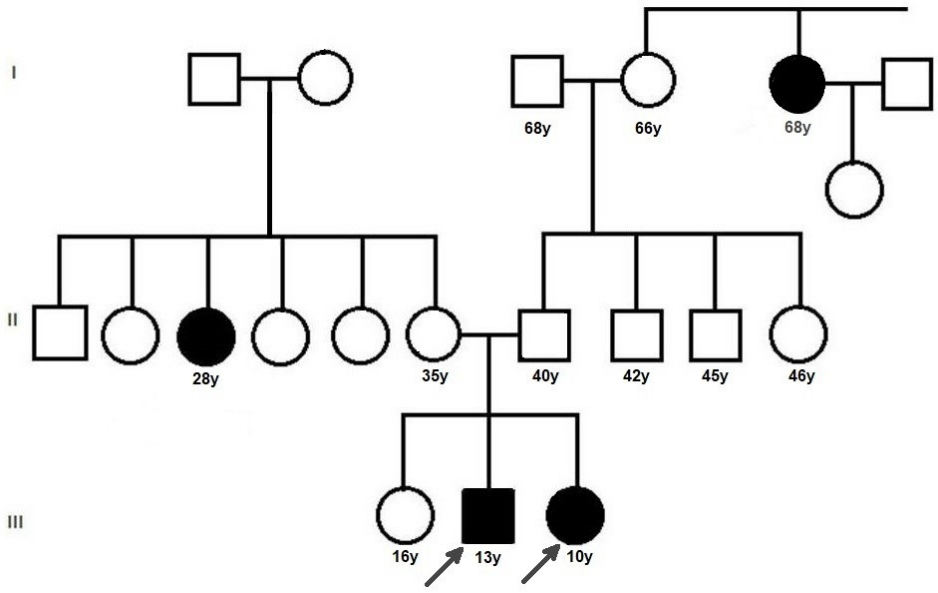
Pedigree chart of 3 generations showing the index cases in generation III, with one each of the mother’s siblings and paternal grandmother’s siblings also affected

The subjects were the 2^nd^ and 3^rd^ offspring of a non-consanguineous married couple. One each of the mother’s siblings and paternal grandmother’s siblings also had a similar disorder. A pedigree chart was traced (Figure 5 (Fig. 5)). The children were referred to a paediatrician for systemic examination and investigations, which showed no internal syndromic associations of the ocular condition of the probands. The eldest sibling did not have any ophthalmologic or internal clinical signs.

Spectacle correction was prescribed to both children along with low-vision aids in the form of magnifying glasses. Ultraviolet-blocking photochromatic lenses or powered sunglasses were requested and skin protection advised. Guarded visual prognosis was explained to the parents. Surgical correction of strabismus/nystagmus was discussed with the parents but they declined consent. They were asked to follow up 6-monthly. Genetic counselling was also done. Genetic testing could not be performed due to non-availability of facility for the same in the institution or nearby, as well as financial constraints.

## Discussion

Our patients exhibit typical features of non-syndromic OCA [1], [5]. OCA is characterized by the systemic findings of hypopigmented skin, hair, eyelashes and eyebrows in addition to the ocular findings. The latter range from nystagmus which is usually pendular and horizontal, poor visual acuity (6/60 or less), loss of stereopsis, intense photophobia, strabismus, large angle kappa, hypochromatic irides with transillumination defects giving the appearance of light blue to almost pink eyes, decreased pigmentation of the retinal pigment epithelium, to the ab-sence or poor definition of foveal pit [1], [3]. Abnormal course of retinal vessels along optic disc is often noted, consisting of an initial nasal deflection followed by an abrupt divergence and reversal of direction to form the temporal vascular arcades [6].

All types of OCA are inherited as autosomal recessive disorders. Parents of an affected child are obligate carriers, and offspring of an affected person are obligate carriers. Recurrence risk for another affected child is 25%; in our case, the siblings are consecutive children. Carriers are asymptomatic. Healthy siblings are at 67% risk of being carriers; hence genetic counselling was done for the eldest sibling as well [1]. In most cases, there is no previous family history of albinism. In our case, we were able to trace a detailed pedigree chart and determine that there was no history of consanguinity. Persons with OCA have normal fertility, and consanguineous marriages can be detrimental as they could propagate the disorder to the next generation. We could not perform a genetic analysis for our patients due to lack of accessible genetic testing facilities as well as financial constraints. This is a limitation of the current case report. It has been shown in an Indian study that genetic testing is helpful in informed decision-making for participating carrier families [7]. 

As regards the management and prognosis of patients of OCA, refractive correction and visual aids should be provided to such patients, especially children at schools in forms of high contrast written material, large type textbooks, optical devices such as magnifiers, large monitors, computer software and smartphone-based assistive apps for the visually impaired [8]. Protection from ultraviolet radiation is also necessary as they have a higher tendency to develop cutaneous malignancies. OCA patients have a normal lifespan with no increase in risk of medical problems as compared to general population except for skin malignancies, which require regular check-ups. Development and intelligence are normal, and well-rehabilitated children are able to lead otherwise healthy and productive lives.

## Conclusion

This report thus represents a typical presentation of oculo-cutaneous albinism in two siblings. Genetic counselling is essential for promoting informed choices, adaptation to individual risks and assessing disease occurrence in the family. Affected children suffer severe visual disability while early identification may potentially mitigate it, hence there is a need to sensitize primary care practitioners regarding general symptoms of OCA.

## Notes

### Patient consent

Patient consent to publish this case report with full face photographs and ocular imaging was obtained.

### Competing interests

The authors declare that they have no competing interests.

## Figures and Tables

**Table 1:**
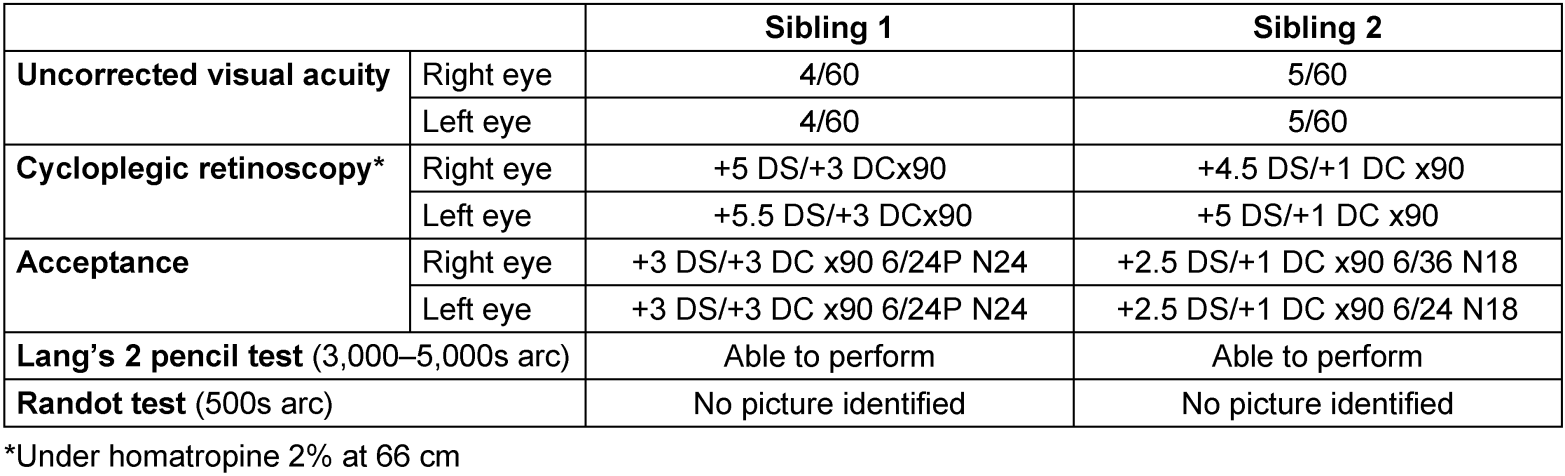
Visual acuity, refraction and stereoacuity tests of our subjects
